# Primary Sjögren's Syndrome: health experiences and predictors of health quality among patients in the United States

**DOI:** 10.1186/1477-7525-7-46

**Published:** 2009-05-27

**Authors:** Barbara Segal, Simon J Bowman, Philip C Fox, Frederick B Vivino, Nandita Murukutla, Jeff Brodscholl, Sarika Ogale, Lachy McLean

**Affiliations:** 1 Associate Professor, Division of Rheumatic and Autoimmune Diseases, Department of Medicine, University of Minnesota, USA; 2 Consultant Rheumatologist, University Hospital Birmingham, UK; 3 Visiting Scientist, Department of Oral Medicine, Carolinas Medical Center, Charlotte, USA; 4 Clinical Associate Professor, Penn Presbyterian Medical Center, USA; 5 Research Manager, Health Care and Policy Research, Harris Interactive, USA; 6 Research Methodologist, Harris Interactive, USA; 7 Health Economist, Genentech, USA; 8 Genentech, USA

## Abstract

**Objective:**

To assess the health related quality of life of patients with primary Sjögren's Syndrome (PSS) in a large US sample.

**Methods:**

Questionnaires were mailed to 547 patients with a confirmed diagnosis of PSS (PhysR-PSS) and all active members of the Sjögren's Syndrome Foundation USA (SSF-PSS), half of whom identified a friend without PSS to also complete the survey.

**Results:**

277 PhysR-PSS patients were compared to 606 controls. The mean age was 62 years in the PhysR-PSS group and 61 years in the control group. 90% in both groups were women. Time from first symptom to diagnosis of PSS was a mean of 7 years. Sicca related morbidity, fatigue severity, depression and pain (assessed by validated questionnaires, PROFAD-SSI, FACIT-F, CES-D, BPI) were significantly greater, and all eight SF-36 domains were significantly diminished, in patients compared to controls. Somatic fatigue was the dominant predictor of physical function and of general health. Depression was the dominant predictor of emotional well being. Health care utilization was higher in patients than controls, including out of pocket dental expenses (mean: PhysR-PSS = $1473.3, controls = $503.6), dental visits (mean: PhysR-PSS = 4.0, controls = 2.3), current treatments (mean: PhysR-PSS = 6.6, controls = 2.5), and hospitalizations (53% PhysR-PSS, vs. 40% controls).

**Conclusion:**

Diminished health quality and excess health costs are prevalent among PSS patients. Health experiences and functional impact of PSS is similar among US and European patients. Delayed diagnosis, sicca related morbidity, fatigue, pain and depression are substantial suggesting unmet health needs and the importance of earlier recognition of PSS.

## 

Primary Sjögren's syndrome (PSS) is a chronic, systemic autoimmune disorder characterized by inflammation of exocrine glands and functional impairment of the salivary and lachrymal glands [1]. Females are affected nine times more frequently than males. Previous European studies have demonstrated significant reductions of health-related quality of life in PSS patients, [2-8]. However, despite the fact that PSS is a common disorder which significantly impacts health status, the effect of PSS on a broad spectrum of quality of life domains including economic resources, work status, leisure activities and inter-personnel relationships has not been well studied. Because PSS is predominantly diagnosed in peri-menopausal women, there is very limited data concerning the health status of younger women particularly those of child bearing age, as well as limited data concerning the health status of men with PSS.

While earlier studies have concluded that PSS is a condition that affects patients physically, psychologically and socially, the factors contributing to diminished health quality in PSS are not well understood. A small study by Sutcliffe demonstrated that with the exception of oral damage, end organ damage was uncommon in primary SS, yet the degree of functional disability was as great in patients with primary SS as in those with SLE[3]. Strombeck investigated health related quality of life in 42 Swedish women with PSS[5]. All 8 scales of the SF-36 were significantly decreased and the percentage of patients not working due to disability was similar among patients with PSS, RA and Fibromyalgia. PSS patients tended to score worse on the psychological scales and experienced better physical function than the RA patients, while the fibromyalgia patients experienced lower levels of health quality on all 8 SF-36 scales compared to both patients with RA and PSS. To date, the relative effects of pervasive symptoms including fatigue, pain, psychological distress and xerostomia on health quality are sparsely documented in the PSS literature; hence the precise causes of diminished functioning in PSS remain unclear.

A recent study of 111 patients with PSS by Champey emphasized the importance of the psychological dimension on results of the SF-36. Fatigue and pain, but not dryness, were correlated with both quality of life and psychological distress[7]. There is limited and inconsistent data regarding the impact of sicca symptoms on health quality. Small sample size and differences in the assessment instruments used make this data difficult to interpret[9,10]. The present study was designed to address these gaps in the PSS literature and to provide a systematic investigation of the health experiences of a large cohort of PSS patients in the United States. In order to provide a comprehensive picture of the health status of PSS patients, data was collected on multiple aspects of health quality including resource utilization, out of pocket expenses, and employment status as well as clinical manifestations.

## Methods

### Overview

Sjogren's patients were recruited through multiple sources to create a representative sample. In addition to a physician-verified cohort of patients (our core sample), we also created a large comparison group of Sjogren's patients for cross-validation purposes by recruiting all active patient members of the Sjogren's Syndrome Foundation (SSF). 'Healthy' controls were recruited through the SSF patients through a process of peer nomination. Data were collected through a mail survey between January 1 and July 31, 2007. Survey responses were anonymous. The study received approval from the Western Institutional Review Board (IRB), and where appropriate, the local IRB's with which the referring physicians' clinics were associated.

### Sample

PSS patients with a confirmed diagnosis according to the 2002 AECG criteria [11] were recruited through nine high-volume clinics across the United States (referred to as "PhysR-PSS"). To protect the patient's identity, the surveys were sent to the physician offices for distribution among eligible patients. We asked the nine physicians to identify from their records all patients classified as having PSS according to the 2002 AECG criteria. We asked physicians who had 100 or fewer patients with PSS to recruit all eligible patients for the survey. We asked physicians who had more than 100 eligible patients to select 100 patients at random and recruit them for the study. Subjects recruited through the Sjogren's foundation (SSF-PSS) were classified as "possible" PSS if they reported a diagnosis of Sjogren's syndrome and history of a positive minor salivary gland biopsy and/or a positive anti-SSA/Ro or anti-SSB/La test result. Questionnaires were also mailed to all active patient members of the Sjögren's Syndrome Foundation ("SSF-PSS"), half of whom were asked to recruit a friend of the same age and gender and without a diagnosis of SS to provide a community control group ("controls"). Patients were specifically instructed not to recruit a relative. Subjects who self-reported a diagnosis of a rheumatic co-morbidity (rheumatoid arthritis, systemic lupus erythematosus, mixed connective tissue disease, myositis or scleroderma) were eliminated from both the patient and the control groups.

### Questionnaire

We devised an extensive health questionnaire: the ASSESS survey (Assessment of Symptoms and Experiences of Sjögren's syndrome) for this study based on recommendations of a panel of Sjogren's investigators. The survey included questions regarding co-morbid conditions and previous health problems that were considered secondary to or possibly associated with Sjogren's. A draft questionnaire was administered to 5 patients with Sjogren's syndrome and revisions were made based on their feedback. No additional validation was undertaken. Key health domains addressed included: frequency and severity of symptoms, diagnostic timeline and path to diagnosis, health conditions experienced, treatments used, provider visits, hospitalizations within the past 5 years, dental visits and costs of dental care as well as the overall impact of Sjogren's on activity, family and social life. The ASSESS questionnaire also included pre-validated instruments for measurement of pain, fatigue, depression and cognitive symptoms. Health related quality of life was assessed with the Medical Outcomes Survey Short Form-36, (SF-36)[12]; pain with the modified Brief Pain Inventory, (BPI)[13]; fatigue with the Functional Assessment of Chronic Illness Therapy, (FACIT-Fatigue)[14], and the Profile of Fatigue and Discomfort-Sicca Symptoms Inventory, (PROFAD-SSI)[8]; cognitive symptoms with the Thinking scale[15] (for more details on this scale see Additional file 1, Table S1); and, depressed mood with the Center for Epidemiologic Studies Depression Scale(CES-D) [16]. The PROFAD-SSI was also used to assess sicca severity.

#### Description of the instruments used

The PROFAD-SSI is comprised of eight domain scores that reflect different manifestations of fatigue and sicca. The domain scores, including somatic and mental fatigue domains, may be used independently or combined into a composite fatigue score (ProF), or further summarized to indices of fatigue and discomfort (PROFAD index) and sicca severity (SSI index). PROFAD domain scores range from 0 to 7 and PROFAD-SSI summary indices range from 0 to 28, with higher scores indicating worse functioning. The BPI is scored into two measures – pain severity and pain interference – that range from 0 to 10 with higher scores indicating worse functioning. The Thinking scale is a 6 item subjective cognitive index. It was developed as part of a disease specific Lupus Qualtiy of life index and while it has been shown to have good internal consistency and test-retest reliability, [15] in a study of 121 subjects with SLE, the index has not been previously validated in primary Sjogren's. The Thinking scale provides a single score that can range from 0 to 100 with higher scores representing poorer functioning. The CES-D scale results in a single score that can range from 0 to 60; a score greater than 16 indicates depression, a score greater than 27 indicates severe depression. The FACIT-F results in a single score that can range from 0 to 52, with higher scores representing better functioning. Each of the eight SF-36 domain scores – physical functioning, emotional well-being, role limitations due to physical functioning/emotional functioning, energy/fatigue, social functioning, pain, and general health – can range from 0 to 100 with higher scores representing better functioning.

### Statistical Methods

#### Group comparisons

Univariate ANOVAs and 2-way chi-square tests were conducted to assess the statistical significance of overall differences between the various participant groups. ANOVAs that were significant (p ≤ 0.05) and that entailed comparisons between three or more groups were followed up with Fisher Least Significant Difference tests to determine which groups were significantly different from one another on the scale or item in question. Similarly, chi-square tests that were significant (p ≤ 0.05) and that entailed comparisons between three or more groups were followed up with separate chi-square tests.

#### Regression Analyses

Linear regression analysis was used to investigate the contribution of sicca symptom severity (SSI), somatic fatigue (domain score from the PROFAD-SSI), mental fatigue (domain score from the PROFAD-SSI), depression (CES-D), pain severity (BPI) and cognitive symptoms (Thinking scale) to quality of life, as defined by the physical functioning, emotional well-being and general health SF-36 domains. Scale scores were used as predictors and age and disease duration were used as covariates. For each dependent variable, the analysis was run two ways, once with each of the critical predictors (i.e. minus age and disease duration) entered into their own regression equations, and once with all of the critical predictors and covariates entered into a single multivariate regression model. The regression analyses were run for each of the two PSS patient groups and for the controls. An effect was said to be reliable if the parameter estimate for the associated predictor was significant by a 2-tailed t-test at p < .05.

## Results

Of the 547 surveys sent to investigators' practices, 281 (51%) were returned completed. Four surveys were identified as duplicates and excluded; analyses were therefore based on 277 PhysR-PSS patients. Of the 8, 694 surveys mailed to SSF members, 3,939 (45%) were returned completed and of these 1,225 were classified as 'possible' PSS according to the eligibility criteria. Since the diagnosis of PSS could not be directly confirmed in the SSF patient group, these data were not included in the analysis, however we found that the clinical characteristics and demographics were, in almost all respects similar, to the verified PSS patients identified through the investigators' practices and to previously reported referral based cohorts. It is of interest therefore to compare the data from the large community based SSF patient sample to the investigator referred sample and we have provided this analysis in the additional section (see Additional file 2, Tables S1 – S5). 630 surveys were received from non-SS controls. Of these, 24 were excluded from the control group as they had reported a diagnosis of SS or another rheumatic condition, leaving 606 non-SS controls.

### Demographic and Clinical Characteristics

Demographics and clinical characteristics of the patient and control groups are displayed in Table 1. The mean time from first symptom to diagnosis was 7 years for PhysR-PSS patients. Ocular and oral dryness were the most common presenting symptoms reported by 44% and 39%, respectively, of PhysR patients; detailed data regarding presenting symptoms has been described previously[17]. Key extra-glandular features such as Raynaud's, purpura, lymph node swelling or pain, and leucopenia were frequently experienced by PhysR-PSS patients. Morbidity related to severe longstanding oral and ocular dryness was significantly greater in patients compared to controls (Table 1). The community-based SSF-PSS group demonstrated similar data albeit with a slightly higher female percentage (93%) and a lower frequency of vasculitis, CNS Sjögren's and Lymphoma than the PhysR-PSS sample (see Additional file 2, Table S1).

**Table 1:** Patient profile: Demographics and clinical features

**Demographics and Clinical Characteristics**	**PhysR-PSS**	**Controls**
	
	N = 277	N = 606
Age (Mean ± S.D.)	62 ± 12.6	61 ± 12.2
Gender (% Female)	90%	92%
Employment Status		
Employed (net)	38%	49% (1,2)
Not Employed (due to disability)	12%*	0%
Disease Duration (Mean ± S.D.)	9.0 ± 8.4	N/A
Time from first symptom to diagnosis (Mean ± S.D.)	7.1 ± 9.4	N/A
Extra-glandular Symptoms		
Raynaud's	51%*	14%
Forgetfulness	67%	62%
Depression (reported by patient)	54%*	41%
Lymph node pain or swelling	41%*	12%
Muscle pain	60%*	42%
Joint pain	78%*	52%
Neuropathy ("pins and needles," tingling and/or numbness in extremities)	70%*	41%
Extra-glandular Conditions		
Purpura/petechia	14%*	4%
Vasculitis	17%*	2%
CNS Sjögren's	22%*	1%
Leucopenia	21%*	5%
Lymphoma	12%*	2%
Lung Disease	16%*	6%
Ocular Sicca-related Disorders		
Chronic blepharitis	30%*	5%
Corneal scarring	18%*	2%

* p < .05.

The impact of Sjogren's syndrome on health related quality of life was substantial. PSS patients were more likely than the non-SS adults to not be working due to disability (Table 1). PhysR-PSS patients reported significant reductions in all eight domains of the SF-36 (Table 2). Additionally, pain, fatigue, depressed mood and cognitive symptoms were significantly greater in patients compared to controls. Depression (CES-D = 16) was present in 37% of patients compared to 12% of controls. In order to assess the impact of gender and disability, and due to the small samples of men and work disabled in the PhysR patient group, the PhysR and SSF patients were combined in analyses comparing men with women and the work disabled with the employed (see Additional file 2, Table S3). Gender did not have a statistically significant effect on any of the psychometric ratings. Patients who were unemployed due to disability reported significantly more pain, depression and cognitive dysfunction than those who were employed (all p values < 0.05).

**Table 2:** Severity/Impact of disease

**Scores on Prevalidated Instruments**	**PhysR-PSS**	**Controls**
	
	N = 277	N = 606
SF-36^***$***^		
Physical Functioning	61.1	81.1 *
Role limitations – Physical	35.0	78.0 *
Role limitations – Emotional	58.1	86.3 *
Energy/Fatigue	38.9	62.2 *
Emotional Well-being	69.4	78.5 *
Social Functioning	65.2	87.6 *
Pain	53.4	77.0 *
General Health	45.5	72.6 *
PROFAD – SSI^**@**^		
PROF	5.3 *	1.9
PROFAD	10.1 *	3.6
SSI	11.7*	3.0
FACIT – Fatigue^***$***^	30.1	43.0*
Modified BPI-SF^**@**^		
Pain Severity	3.9*	1.5
Pain Interference	3.3 *	1.0
CESD^**@**^	14.9 *	7.7
Thinking^@^	30.1 *	16.4

^$ ^Higher scores indicate better functioning; ^@ ^Higher scores indicate worse functioning

* p < .05.

### Health Care Utilization

Health care utilization among PSS patients was high. PSS patients were significantly more likely than controls to have been hospitalized in the past 5 years (PhysR-PSS = 53%, controls = 40%, *p *< 0.05). They experienced more frequent infections, including urinary tract (PhysR-PSS = 44%, controls = 37%, *p *< 0.05), pneumonia (PhysR-PSS = 32%, controls = 23%, *p *< 0.05), and vaginal infections (PhysR-PSS = 38%, controls = 29%, *p *< 0.05). Differences in health care provider visits were largely accounted for by visits to a rheumatologist (PhysR-PSS = 94%, controls = 13%, *p *< 0.05), ophthalmologist (PhysR-PSS = 79%, controls = 51%, *p *< 0.05) or a neurologist (PhysR-PSS = 49%, controls = 16%, *p *< 0.05). PSS patients were also more likely than controls to use multiple medications (mean number of medications both prescription and over the counter medications) currently taken: PhysR-PSS = 6.7, controls = 2.5, *p *< 0.05). Out-of-pocket spending for dental care was two to three fold higher in the patient group compared to the peer group (mean out-of-pocket spending in the past year: Phys R-PSS = $1473.30; controls = $503.60, p < 0.05.

### Predictors of Health-Related Quality of Life

**Figure 1:**
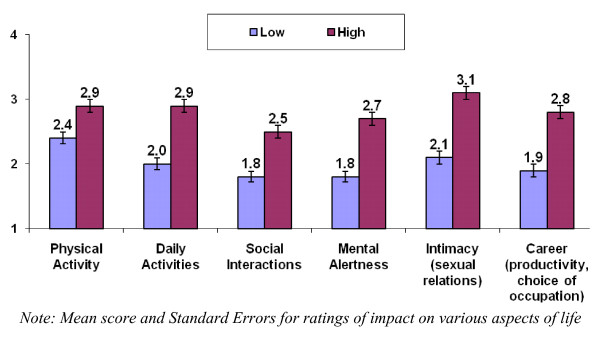
**Impact of SS among patients with low and high sicca severity**.

Respondents were asked to rate the impact of SS (or their "health" if non-SS controls) on various aspects of their life on four point scales, where 1 indicated no impact and 4 indicated a major impact. Patients reported a greater impact on multiple aspects of their lives than controls, including physical activity (PhysR-PSS = 2.6, controls = 1.8, *p *< 0.05), intimacy (PhysR-PSS = 2.5, controls = 1.5, *p *< 0.05), career (PhysR-PSS = 2.3, controls = 1.3, *p *< 0.05), daily activities (PhysR-PSS = 2.4, controls = 1.4, *p *< 0.05), social interactions (PhysR-PSS = 2.1, controls = 1.3, *p *< 0.05) and mental alertness (PhysR-PSS = 2.2, controls = 1.3, *p *< 0.05). Patients with more severe sicca symptoms reported significantly greater impact of Sjogren's syndrome on all activities (Figure 1).

The results of the multivariate regression analysis are shown in Table 3. Among the PhysR-PSS, sicca severity and disease duration were not significant contributors to impaired quality of life in any of the full models (with age and disease duration taken into account). Somatic fatigue was the only unique predictor of general health; pain severity and depression were the only unique predictors of emotional well-being, and physical functioning was predicted by age, pain severity and somatic fatigue. For emotional well-being, the dominant unique predictor of quality of life was depression, accounting on its own for 25% of the variance in the index among the PhysR-PSS patients. The contributions of both depression and fatigue are substantial and contribute uniquely to various aspects of disability.

**Table 3:** Multiple linear regression model of health quality in PSS patients and controls

	**SF-36 Domain Dependent Variable**								
	
**Predictor**	**Physical Functioning**			**Emotional Well-Being**			**General Health**		
	
	***R***^**2**^	***β***	***SE***	***R***^**2**^	***β***	***SE***	***R***^**2**^	***β***	***SE***
**PhysR-PSS Patients (*n *= 201):**									
Sicca severity (domain from SSI)	0.01	0.09	0.32	0.00	0.03	0.19	0.00	-0.06	0.23
Somatic fatigue (domain from PROFAD-SSI)	0.07	-0.52***	1.44	0.01	-0.17	0.87	0.08	-0.54***	1.05
Mental fatigue (domain from PROFAD-SSI)	0.00	-0.03	1.34	0.00	-0.03	0.81	0.00	-0.03	0.98
Depression (CES-D)	0.00	0.01	0.19	0.25	-0.72***	0.11	0.01	-0.12	0.14
Pain severity (domain from BPI)	0.04	-0.28***	0.83	0.02	0.18**	0.50	0.00	-0.07	0.60
Age	0.05	-0.25***	0.13	0.00	0.05	0.08	0.01	0.09	0.10
Duration of disease	0.00	-0.04	0.20	0.00	0.04	0.12	0.00	-0.02	0.14
									
**Controls (*n *= 498):**									
Sicca severity (domain from SSI)	0.00	0.09*	0.28	0.00	-0.01	0.18	0.01	-0.10*	0.24
Somatic fatigue (domain from PROFAD-SSI)	0.05	-0.37***	0.98	0.00	0.00	0.63	0.08	-0.46***	0.85
Mental fatigue (domain from PROFAD-SSI)	0.00	0.08	0.90	0.00	-0.07	0.58	0.01	0.13*	0.79
Depression (CES-D)	0.00	0.01	0.12	0.25	-0.65***	0.08	0.01	-0.13**	0.10
Pain severity (domain from BPI)	0.08	-0.35***	0.52	0.00	-0.02	0.34	0.01	-0.15***	0.46
Age	0.11	-0.34***	0.06	0.04	0.19***	0.04	0.00	-0.05	0.05

*Note*: The R-square values for the individual predictors are the incremental R-squares.

*Note*: All coefficients marked with one or more asterisks are significant by a two-tailed *t*-test.

* *p *< .05; ** *p *< .01; *** *p *< .001.

### PROFAD-SSI

**Figure 2:**
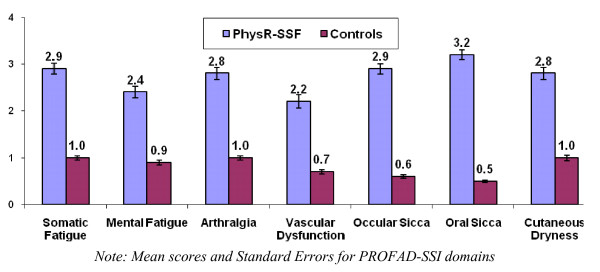
**Ratings of fatigue and sicca severity on PROFAD-SSI domains among PhysR patients and controls**.

We and others have previously carried out validation of this questionnaire in European patients with PSS, RA and SLE [5,18,19] but not in the USA. As indicated in Figure 2, the PROFAD-SSI distinguished between patients and controls on all domains of the scale: PSS patients reported more somatic fatigue, mental fatigue, arthritic symptoms, uncomfortably cold hands, oral dryness, ocular dryness, cutaneous dryness and vaginal dryness, all *t*s > 12.0, all *p*s < 0.001. Similar data was obtained using the summary PROF, PROFAD and SSI indices. Principal component analysis was used to investigate the internal structure of the PROFAD-SSI. Facet scores rather than individual items were used for ease of interpretation and presentation but gave similar results individual items (see Additional file 3, Tables S1 – S3). Aggregation of the individual PROFAD items into facet scores was supported by reliability analyses, which showed each of the facets to be highly consistent internally (range of Cronbach's alpha values: 0.74 to 0.97).

## Discussion

This is the first study to investigate health status in a large cohort of PSS patients in the US and is the most comprehensive description to date of the burden of illness experienced by PSS patients. We documented reduced functioning among PSS patients in every domain of the SF-36, and increased utilization of health care services including medications, hospitalization rates, provider visits and out- of pocket expenses. Compared to their peer controls, PSS patients also reported greater work disability.

The comparison to age and gender-matched controls suggests that the symptoms experienced by PSS patients are related to the disease and not attributable to natural processes of aging. Our data demonstrates that the reduction in health-related quality of life in PSS is similar to that experienced by patients with RA and SLE[3,5,20]. The reduction in quality of life experienced by PSS patients in our cohort is highlighted by comparison of the fatigue score, as measured by the FACIT-F, and the physical function component score of the SF-36 to that recently reported in a large cohort of Rheumatoid Arthritis patients with active disease who had failed anti-TNF therapy[21] The FACIT-F of 30.33 and the SF-36 role limitations, physical = 30.9 reported in the active RA group are strikingly similar to the scores of 30.1(FACIT-F) and 35.0 (SF-36 role limitations, physical) in our PSS cohort.

The characteristics of the patient population in terms of mean age, gender and duration of symptoms were similar to that previously reported in European PSS cohorts [7,22]. The mean time to diagnosis of 7 years in our patients is not unusual for PSS possibly due to the nonspecific nature of the presenting symptoms or to poor physician awareness of PSS in general and confirms that, despite a growing quantity of research regarding the severity of sicca symptoms and range of extra-glandular manifestations, recognition of the diagnosis is typically delayed for many years after the onset of sicca symptoms. Moreover, while we recognize the limitations of self-reported data, the patients identified by experienced physician investigators in this study as meeting current American European Consensus Criteria for diagnosis gave highly similar information in almost every category including rates of ocular and oral complications, key extra-glandular features such as Raynaud's, joint swelling, purpura, lung disease, and lymphoma to that previously reported in European cohorts[6,23]. The validity of this sample is supported by the expected prevalence of dry eye and dry mouth, cardinal features of the disorder which are highly predictive of the diagnosis. The generalizability of the findings in the PhysR-PSS group is additionally supported by the highly similar parallel health data provided by the larger SSF-PSS group (see Additional file 2, Table S1 – S5).

Morbidity related to sicca symptoms was high. On both the SF-36 and in the impact questions, patients with greater sicca severity did report poorer functioning (see Additional file 2, Table S1 – S5). However, sicca severity did not contribute uniquely to health quality in any of the multivariate models.

Cultural differences between countries do not appear to contribute to health quality among patients with PSS. The functional impact of PSS in our patients is similar to that previously reported in European cohorts[4,5,7,8] Subjective memory loss and concentration difficulties are commonly reported by PSS patients but do not contribute to disability independently of depression.

The relatively high rate of psychological and cognitive symptoms reported by patients in our cohort is consistent with data in the literature suggesting an increase in affective disorders in PSS [24-27]. Our data on the prevalence of depression in PSS is consistent with that of previous studies. Valtysdottir measured psychological status in a Swedish cohort (N = 67) of PSS patients and reported that possible depression was present in 30%, that 42% experienced symptoms of anxiety and 60–70% reported cognitive dysfunction[28]. In another study, by the same group of investigators, it was also concluded that patients with PSS have more psychiatric symptoms and lower sense of well-being than patients with RA [29]. Despite somewhat different case selection criteria and use of different instruments, similar findings of increased psychological distress in SS have been a consistent finding between countries and among different cultural groups [30-34]

While the PSS subjects reported significantly greater depression and cognitive symptoms than controls, mental fatigue (memory problems and difficulty concentrating) was not a unique predictor in any of the multivariate models. The relationship between the depression frequently encountered in PSS patients and cognitive impairment is not well understood. Fatigue and depression, along with pain, anxiety and sleep impairment can lead to abnormal cognition. It is also the case that patients with depression over estimate the degree of cognitive dysfunction they are experiencing[35]. More data is needed on the relationship between subjective cognitive function and cognitive performance in SS patients. It is possible that depression and cognitive impairment are independent but overlapping manifestations of central nervous system disorder in SS patients. A recent community based study[36] in which SLE patients were compared to those with Primary Sjogren's syndrome found similar rates of headaches (87% vs.78%), cognitive dysfunction 46% vs. 50%) and mood disorders (26% vs 33%). The lower incidence of CNS Sjogren's (14–22%) reported by participants in this survey suggests that the common complaints of headache, depression and symptoms of cognitive dysfunction are not usually diagnosed as CNS Sjogren's.

Patients who reported work disability also reported more frequent cognitive symptoms, as well as fatigue, pain and depression. However, factors predictive of work disability have not been examined previously in PSS, and it is unknown whether cognitive impairment contributes to work disability as has been shown previously in systemic lupus[37].

Our study design does have limitations pertaining to sampling methodology. No information was available from non-responders nor could non responders be recontacted as data was collected anonymously. However, the response rate of 50% among the patient samples is considered excellent for blind mailed surveys. Reliance on self-report data is also a weakness of this study however in general, all demographic and clinical data are in agreement with earlier findings. Further research is needed to confirm the impact of PSS on health resources and employment suggested by our data.

In summary, this survey of the health experiences reported by PSS subjects suggests a large unmet health burden. Delays in the diagnosis of Sjogren's syndrome may contribute to the psychological distress of unexplained symptoms and prevent the timely application of symptomatic therapies that are effective in preventing sicca related complications. Earlier diagnosis could potentially reduce morbidity attributable to sicca complications such as corneal scarring and tooth loss. Improved understanding of the neurobiology of pain and fatigue, as well as greater appreciation of the pervasive effects and reduced quality of life experienced by patients with PSS, is needed to reduce the health care costs and ultimately the burden of illness experienced by those with PSS.

## Competing interests

The Medical Authors of this paper received consultancy payments from Genentech for their time spent on questionnaire and project design, project implementation and data analysis in preparation for publication.

## Authors' contributions

BS participated in the design of the study, proposed the focus of this paper, and drafted this manuscript. SJB participated in the design of the study, directed the principal components analyses described in this paper and in the supplementary section, and drafted sections of this manuscript. PCF participated in the design of the study. FV participated in the design of the study. NM participated in the design of the methodology, managed data collection, conducted some of the analyses, and drafted sections of this manuscript. JCB conducted the multivariate regressions and the principal components analyses described in this paper, and drafted some of the results described in this paper. SO participated in the design of this study. LM participated in the design of this study. All authors read and approved the final manuscript.

## Supplementary Material

Additional file 1 T**he thinking scale**. A description of Thinking Scale and its scoring. Click here for file

Additional file 2 **Comparison of physician office patients with patients recruited through the Sjogren's Syndrome Foundation**. The data provided represent the statistical comparison of data from the physician-office patients with the patient controls recruited via the Sjogren's Syndrome Foundation. Click here for file

Additional file 3 **Validation of the PROFAD-SSI among Sjogren's syndrome patients in the United States**. The data provided represent the statistical validation of the PROFAD-SSI among Sjogren's syndrome patients in the United States. Click here for file
