# Supplementary material for: Primary Sjögren's Syndrome: health experiences and predictors of health quality among patients in the United States
Source: Health Qual Life Outcomes. 2009 May 27;7:46. doi: 10.1186/1477-7525-7-46 (PMC2693523; doi:10.1186/1477-7525-7-46)
Supplement: Additional file 1 — The thinking scale. A description of Thinking Scale and its scoring. [file 1477-7525-7-46-S1.doc]

**Table S1. The Thinking/ Cognitive Scale**

By circling one (1) number per line, please indicate whether each activity may have been a problem over the past 4 weeks:

|  | Never a  problem | A problem  some of  the time | A problem  most of the  time | A problem all of  the time—  Unable to do |
| --- | --- | --- | --- | --- |
| 1. Remember details of your recent experiences? | 0 | 1 | 2 | 3 |
| 2. Remember details at home or work? | 0 | 1 | 2 | 3 |
| 3. Concentrate on a task you need to do? | 0 | 1 | 2 | 3 |
| 4. Concentrate on more than one task a time? | 0 | 1 | 2 | 3 |
| 5. Concentrate on reading a book or newspaper? | 0 | 1 | 2 | 3 |
| 6. Find the correct word during conversations? | 0 | 1 | 2 | 3 |

The Thinking scale consists of 6 items selected from the 21-item Cognitive Symptom Inventory (CSI) developed by Pincus et al.^1^ The items were selected based on concepts identified through lupus patient focus groups. The scale has not been published and the psychometric analyses are published in the form of an abstract.^2^ In lupus patients, the scale was found to have good internal consistency (Cronbach’s α =0.91) and test-retest reliability (0.86). In principal components analyses the 6 items loaded on a single factor. The scale had moderate but significant correlations with the SLEDAI, Patient and Clinician Global Impression of disease activity, domains of the SF-36, the Brief Pain Inventory and the FACIT-Fatigue score.

References:

1. Pincus T SC, Callahan LF: A self-report cognitive symptoms inventory to assess patients with rheumatic diseases: results in eosinophilia-myalgia syndrome (EMS), Fibromyalgia, rheumatoid arthritis, and other rheumatic diseases (abstract). Arthritis Rheum 29:S261, 1996

2. Yu EB, Shikiar R, Howard K, Kalunian KC, Petrillo J, Thompson C, et al. Validation of LUP-QOL: a lupus-specific measure of health-related quality of life (HRQL) [abstract]. Ann Rheum Dis 2006;65 Suppl II:601.
