# Supplementary material for: Primary Sjögren's Syndrome: health experiences and predictors of health quality among patients in the United States
Source: Health Qual Life Outcomes. 2009 May 27;7:46. doi: 10.1186/1477-7525-7-46 (PMC2693523; doi:10.1186/1477-7525-7-46)

| **Demographics and Clinical Characteristics** | **Phy.-Ref. PSS** | **SSF-PSS** | **Controls** |
| --- | --- | --- | --- |
|  | **N = 277** | **N = 1,225** | **N = 606** |
|  | (A) | (B) | (C) |
| ***Age*** (Mean ± S.D.) | 62 ± 12.6 | 61 ± 12.7 | 61 ± 12.2 |
| ***Gender*** (% Female) | 90% | 93% ^AC^ | 92% |
| ***Employment Status*** |  |  |  |
| Employed (net) | 38% | 41% | 49% ^AB^ |
| Not Employed (due to disability) | 12% ^BC^ | 8% ^C^ | 0% |
| ***Disease Duration*** (Mean ± S.D.) | 9.0 ± 8.4 | 10.1 ± 8.2 | N/A |
| ***Time from first symptom to diagnosis***  (Mean years ± S.D.) | 7.1 ± 9.4 | 7.0 ± 8.7 | N/A |
| ***Mode of presentation (Top 4)*** |  |  |  |
| Dry Eyes | 44% | 41% | N/A |
| Dry Mouth | 39% | 34% | N/A |
| Fatigue | 27% | 23% | N/A |
| Muscle Pain | 21% ^B^ | 12% | N/A |
| ***Extra-glandular Symptoms*** |  |  |  |
| Raynaud’s | 51% ^C^ | 45% ^C^ | 14% |
| Forgetfulness | 67% | 74% ^AC^ | 62% |
| Depression (reported by patient) | 54% ^C^ | 55% ^C^ | 41% |
| Lymph node pain or swelling | 41% ^C^ | 44% ^C^ | 12% |
| Muscle pain | 60% ^C^ | 69% ^AC^ | 42% |
| Joint pain | 78% ^C^ | 77% ^C^ | 52% |
| Neuropathy (“pins and needles,” tingling and/or numbness in extremities) | 70% ^C^ | 69% ^C^ | 41% |
| ***Extra-glandular Conditions*** |  |  |  |
| Purpura/petechia | 14% ^C^ | 19% ^C^ | 4% |
| Vasculitis | 17% ^BC^ | 10% ^C^ | 2% |
| CNS Sjögren's | 22% ^BC^ | 14% ^C^ | 1% |
| Leucopenia | 21% ^C^ | 24% ^C^ | 5% |
| Lymphoma | 12% ^BC^ | 5% ^C^ | 2% |
| Lung Disease | 16% ^C^ | 15% ^C^ | 6% |
| ***Sicca-related Disorders*** |  |  |  |
| Loss of teeth | 37% ^C^ | 32% ^C^ | 19% |
| Yeast (fungal) infections in the mouth | 31% ^C^ | 35% ^C^ | 4% |
| Severe gum disease | 21% ^C^ | 18% ^C^ | 6% |
| Chronic blepharitis | 30% ^C^ | 43% ^AC^ | 5% |
| Corneal scarring | 18% ^BC^ | 13% ^C^ | 2% |
| Salivary Stones | 17% ^C^ | 20% ^C^ | 3% |

**Table S1. Patient Profile: Demographics and Clinical Features**

*Superscripts indicate that the data in the cell is significantly higher than the data in the cell(s) referred to, based on significance of < .05. Differences between groups in mean values were tested with Univariate ANOVAS and follow-up t-tests. Differences in percentages were tested with Chi-square tests.*

**Table S2. Symptom Severity and Impact of Sjogren’s Syndrome on Health-Related Quality of Life**

| **Scores on Prevalidated Instruments** | **Phy.-Ref. PSS** | **SSF-PSS** | **Controls** |
| --- | --- | --- | --- |
|  | **N = 277** | **N = 1,225** | **N = 606** |
|  | **(A)** | **(B)** | **(C)** |
| ***SF-36**** |  |  |  |
| Physical Functioning | 61.1 | 64.3 | 81.1 ^AB^ |
| Role limitations - Physical | 35.0 | 34.5 | 78.0 ^AB^ |
| Role limitations - Emotional | 58.1 | 66.2 ^A^ | 86.3 ^AB^ |
| Energy / Fatigue | 38.9 | 38.6 | 62.2 ^AB^ |
| Emotional Well-being | 69.4 | 70.8 | 78.5 ^AB^ |
| Social Functioning | 65.2 | 64.5 | 87.6 ^AB^ |
| Pain | 53.4 | 55.1 | 77.0 ^AB^ |
| General Health | 45.5 | 43.8 | 72.6 ^AB^ |
| ***PROFAD - SSI***** |  |  |  |
| PROF | 5.3 ^C^ | 5.4 ^C^ | 1.9 |
| PROFAD | 10.1 ^C^ | 10.4 ^C^ | 3.6 |
| SSI | 11.7 ^C^ | 12.6 ^AC^ | 3.0 |
| ***FACIT - Fatigue**** | 30.1 | 29.8 | 43.0 ^AB^ |
| ***Modified BPI-SF***** |  |  |  |
| Pain Severity | 3.9 ^C^ | 3.7  ^C^ | 1.5 |
| Pain Interference | 3.3 ^C^ | 3.2 ^C^ | 1.0 |
| ***CESD***** | 14.9 ^C^ | 14.0 ^C^ | 7.7 |
| ***Thinking***** | 30.1 ^C^ | 31.9 ^C^ | 16.4 |

** Higher scores indicate better functioning;** Higher scores indicate worse functioning*

*Superscripts indicate that the data in the cell is significantly higher than the data in the cell(s) referred to, based on significance of < .05. Differences between groups in mean values were tested with Univariate ANOVAS and follow-up t-tests. Differences in percentages were tested with Chi-square tests.*

**Table S3. Effects of Gender and Employment Status on Symptom Severity and on the SF-36 General Health Domain in PhysR and SSF-PSS patients combined****

| **Health Outcomes** | **Gender** | | | **Employment** | | | |
| --- | --- | --- | --- | --- | --- | --- | --- |
|  | **Male** | **Female** |  | **Not employed, due to disability** | **Not employed (non-disability reasons)** | **Employed (full, part-time, or self-employed)** |  |
|  | **N = 75** | **N = 1392** |  | **N = 129** | **N = 722** | **N = 608** |  |
|  | **(A)** | **(B)** |  | **(C)** | **(D)** | **(E)** |  |
| **SF-36 General Health** | 46.6 ± 23.0 | 44.0 ± 22.2 |  | 26.5 ± 15.9 | 46.7 ± 21.8 ^C^ | 46.0 ± 22.0 ^C^ |  |
| **Pain (BPI)** | 3.4 ± 2.5 | 3.8 ± 2.6 |  | 5.3 ± 2.3 ^DE^ | 3.6 ± 2.5 | 3.5 ± 2.5 |  |
| **Fatigue (FACIT-F)** | 31.8 ± 12.5 | 29.8 ± 12.2 |  | 19.1 ± 9.8 | 31.5 ± 11.7 ^C^ | 30.8 ± 11.9 ^C^ |  |
| **Depression (CES-D)** | 13.7 ± 10.3 | 14.1 ± 9.9 |  | 21.2 ± 11.1 ^DE^ | 13.1 ± 8.6 | 13.4 ± 10.1 |  |
| **Thinking Scale** | 32.0 ± 24.6 | 31.6 ± 19.4 |  | 45.8 ± 20.4 ^DE^ | 28.0 ± 18.0 | 32.1 ± 19.9 ^D^ |  |
| **PROFAD - SSI** |  |  |  |  |  |  |  |
| PROFAD | N/A | 12.5 ± 6.0 |  | 15.5 ± 5.7 ^DE^ | 9.5 ± 5.7 | 9.9 ± 6.2 |  |
| Sicca Severity (SSI) | 8.6 ± 6.2 | 10.4 ± 6.1 ^A^ |  | 15.5 ± 5.8 ^DE^ | 12.2 ± 6.2 | 11.9 ± 5.7 |  |

*Superscripts indicate that the data in the cell is significantly higher than the data in the cell(s) referred to, based on significance of < .05. Differences between groups in mean values were tested with Univariate ANOVAS and follow-up t-tests. Differences in percentages were tested with Chi-square tests.*

** Summary scores cannot be calculated if any of the component scores are missing for a respondent.  Vaginal dryness is one of the components of the SSI, and since men have missing data on this variable, they have missing data on the summary score.*

*** Due to the small samples of men and work disabled in the PhysR patient group, the PhysR and SSF patient groups were combined.*

**Table S4. Comparison of the mean impact of SS on physical activities, intimacy and career among different demographic and clinical groups (SSF and Phy-Ref pSS combined**).**

| **Impact of SS** | **Gender** | |  | **Age at Onset** | | |  | **Sicca Severity** | |  |
| --- | --- | --- | --- | --- | --- | --- | --- | --- | --- | --- |
|  | **Male** | **Female** | ***P*** | **<35** | **35-70** | **>70** | ***P*** | **Low**  **(< 12)** | **High (≥ 12)** | ***P*** |
|  | **N=75** | **N=1396** |  | **N=148** | **N=1252** | **N=74** |  | **N=617** | **N=663** |  |
|  | (A) | (B) |  | (C) | (D) | (E) |  | (F) | (G) |  |
| **Physical Activities (walking, climbing)** | 2.4  ± 1.0 | 2.6  ± 1.0 | 0.12 | 2.4  ± 1.1 | 2.6  ± 1.0 ^C^ | 2.7  ± 1.0 ^C^ | 0.03 | 2.3  ± 1.0 | 2.9  ± 1.0 ^F^ | 0.00 |
| **Intimacy (sexual relations)** | 2.1  ± 1.2 | 2.6  ± 1.1 ^A^ | 0.00 | 2.7  ± 1.1 ^DE^ | 2.5  ± 1.2 | 2.2  ± 1.2 | 0.02 | 2.1  ± 1.1 | 3.0  ± 1.0 ^F^ | 0.00 |
| **Career (productivity, choice of occupation)** | 2.2  ± 1.3 | 2.4  ± 1.2 | 0.34 | 2.5  ± 1.2 ^E^ | 2.4  ± 1.2 ^E^ | 1.5  ± 0.8 | 0.00 | 2.0  ± 1.1 | 2.8  ± 1.2 ^F^ | 0.00 |
| **SF-36 General Health Domain** | 46.6  ± 23.0 | 43.9  ± 22.2 | 0.32 | 41.7  ± 22.2 | 44.1  ± 22.3 | 49.8  ± 21.4 ^CD^ | 0.04 | 51.2  ± 20.6 ^G^ | 37.5  ± 21.4 | 0.00 |
| PROFAD Index | 8.6  ± 6.2 | 10.4  ± 6.2 ^A^ | 0.01 | 10.1  ± 6.2 | 10.4  ± 6.2 | 8.8  ± 5.4 | 0.21 | 7.1  ± 4.7 | 13.5  ± 5.7 ^F^ | 0.00 |
| SSI | N/A | 12.5 ± 6.0 | N/A | 12.3  ± 5.9 | 12.5  ± 6.1 | 11.2  ± 6.2 | 0.31 | 7.2  ± 3.0 | 17.4  ± 3.5 ^F^ | 0.00 |

*Superscripts indicate that the cell is significantly higher than the cell(s) referred to, based on significance < .05. Differences between groups in mean values were tested with Univariate ANOVAS and follow-up t-tests. Differences in percentages were tested with Chi-square*

*** Due to the small samples of men and work disabled in the PhysR patient group, the PhysR and SSF patient groups were combined.*

**Table S5. Multiple Linear Regression Model of Health Quality in PSS**


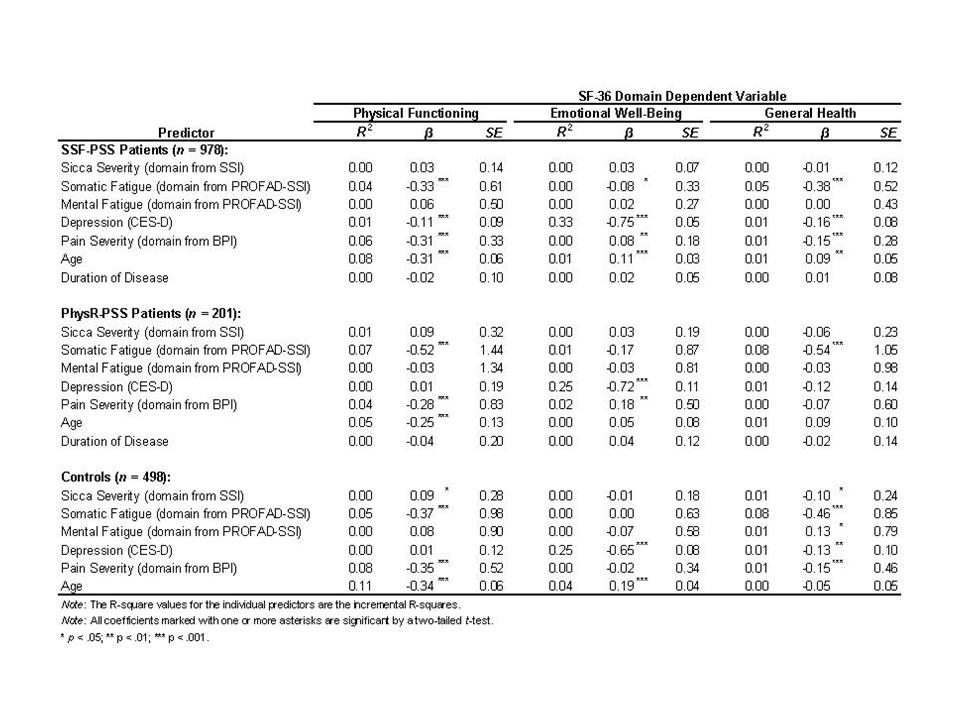

Supplement: Additional file 2 — Comparison of physician office patients with patients recruited through the Sjogren's Syndrome Foundation. The data provided represent the statistical comparison of data from the physician-office patients with the patient controls recruited via the Sjogren's Syndrome Foundation. [file 1477-7525-7-46-S2.doc]
