# Supplementary material for: Primary Sjögren's Syndrome: health experiences and predictors of health quality among patients in the United States
Source: Health Qual Life Outcomes. 2009 May 27;7:46. doi: 10.1186/1477-7525-7-46 (PMC2693523; doi:10.1186/1477-7525-7-46)
Supplement: Additional file 3 — Validation of the PROFAD-SSI among Sjogren's syndrome patients in the United States. The data provided represent the statistical validation of the PROFAD-SSI among Sjogren's syndrome patients in the United States. [file 1477-7525-7-46-S3.doc]

**Table S1.** Factor loadings of the *a priori* scored facets into domain-like groupings (PhysR-PSS patients n=124).

| Components | Domain assigned in the a priori system | Facet Name | Factor loadings on components | | | | | | |
| --- | --- | --- | --- | --- | --- | --- | --- | --- | --- |
|  |  |  | 1 | 2 | 3 | 4 | 5 | 6 | 7 |
| 1 | Somatic Fatigue | Low Stamina | **0.91** | 0.26 | 0.07 | 0.09 | 0.07 | -0.01 | 0.15 |
| 1 | Somatic Fatigue | Poor Starting | **0.88** | 0.21 | 0.09 | 0.07 | 0.16 | 0.07 | 0.16 |
| 1 | Somatic Fatigue | Need Rest | **0.87** | 0.19 | 0.10 | 0.07 | 0.07 | 0.07 | 0.17 |
| 1 | Mental Fatigue | Poor Concentration | **0.85** | 0.13 | 0.27 | 0.14 | 0.10 | 0.22 | -0.15 |
| 1 | Mental Fatigue | Poor Memory | **0.85** | 0.10 | 0.29 | 0.13 | 0.13 | 0.20 | -0.21 |
| 1 | Somatic Fatigue | Weak Muscles | **0.76** | 0.29 | 0.08 | 0.14 | 0.10 | 0.08 | 0.37 |
| 1 | Arthralgia | Painful Limbs | **0.67** | 0.22 | 0.19 | 0.12 | 0.23 | 0.15 | 0.43 |
| 2 | Oral Sicca | Difficulty Eating | 0.16 | **0.90** | 0.09 | 0.06 | 0.15 | 0.08 | 0.07 |
| 2 | Oral Sicca | Dry Throat or Nose | 0.21 | **0.84** | 0.23 | 0.16 | 0.09 | 0.23 | 0.10 |
| 2 | Oral Sicca | Bad Breath | 0.29 | **0.74** | 0.34 | 0.03 | 0.06 | 0.06 | 0.08 |
| 2 | Oral Sicca | Oral Problems | 0.32 | **0.74** | 0.07 | 0.28 | 0.24 | 0.20 | 0.03 |
| 3 | Occular Sicca | Poor Vision | 0.47 | 0.27 | **0.73** | 0.09 | 0.03 | 0.08 | 0.14 |
| 3 | Occular Sicca | Sore Eyes | 0.21 | 0.49 | **0.62** | 0.32 | 0.30 | 0.00 | 0.04 |
| 3 | Occular Sicca | Eye Irritation | 0.09 | 0.52 | **0.60** | 0.25 | 0.31 | 0.18 | 0.10 |
| 4 | Vascular Dysfunction | Uncomfortably cold hands | 0.13 | 0.18 | 0.16 | **0.92** | 0.00 | 0.03 | 0.06 |
| 4 | Cutaneous Dryness | Cutaneous Dryness Domain | 0.42 | 0.22 | 0.12 | **0.50** | 0.36 | 0.09 | 0.35 |
| 5 | Vaginal dryness | Vaginal dryness | 0.25 | 0.31 | 0.18 | 0.05 | **0.85** | 0.06 | 0.11 |
| 6 | Oral Sicca | Wetting Mouth | 0.28 | 0.40 | 0.11 | 0.05 | 0.07 | **0.83** | 0.12 |
| 7 | Arthralgia | Painful Hands | 0.46 | 0.15 | 0.28 | 0.32 | 0.23 | 0.24 | **0.54** |
|  |  |  |  |  |  |  |  |  |  |

**Table S2.** Factor loadings of the *a priori* scored facets into summary indices. PhysR patients n=124.

| Components | Domain assigned in the a priori system | Facet Name | Factor Loadings on Components | |
| --- | --- | --- | --- | --- |
|  |  |  | 1 | 2 |
| 1 | Somatic Fatigue | Low Stamina | **0.91** | 0.24 |
| 1 | Somatic Fatigue | Poor Starting | **0.90** | 0.25 |
| 1 | Somatic Fatigue | Need Rest | **0.88** | 0.21 |
| 1 | Mental Fatigue | Poor Concentration | **0.86** | 0.25 |
| 1 | Mental Fatigue | Poor Memory | **0.85** | 0.23 |
| 1 | Somatic Fatigue | Weak Muscles | **0.81** | 0.34 |
| 1 | Arthralgia | Painful Limbs | **0.76** | 0.38 |
| 1 | Arthralgia | Painful Hands | **0.62** | 0.46 |
| 1 | Cutaneous Dryness | Cutaneous Dryness Domain | **0.54** | 0.49 |
| 1 | Ocular Sicca | Poor Vision | **0.54** | 0.53 |
| 2 | Oral Sicca | Dry Throat or Nose | 0.22 | **0.87** |
| 2 | Ocular Sicca | Eye Irritation | 0.18 | **0.85** |
| 2 | Oral Sicca | Difficulty Eating | 0.13 | **0.83** |
| 2 | Ocular Sicca | Sore Eyes | 0.28 | **0.80** |
| 2 | Oral Sicca | Oral Problems | 0.33 | **0.79** |
| 2 | Oral Sicca | Bad Breath | 0.29 | **0.75** |
| 2 | Vaginal Dryness | Vaginal Dryness | 0.33 | **0.57** |
| 2 | Oral Sicca | Wetting Mouth | 0.36 | **0.55** |
|  | Vascular Dysfunction | Uncomfortably Cold Hands | 0.21 | 0.47 |

**Table S3.** Pearson correlations between the PROFAD and the SF-36 and the FACIT-F fatigue index among PhysR-PSS patients (N=124 evaluable patients)^+^.

| **Item** | **PROFAD Somatic Fatigue Domain** | **PROFAD Mental Fatigue Domain** | **PROFAD Arthralgia Domain** | **PROFAD Uncomfortably cold hands** | **SSI Occular Sicca Domain** | **SSI Oral Sicca Domain** | **SSI Cutaneous Dryness Domain** | **SSI Vaginal dryness** |
| --- | --- | --- | --- | --- | --- | --- | --- | --- |
| SF36 Physical functioning | -0.61* | -0.47* | -0.60* | -0.09 | -0.30* | -0.32* | -0.36* | -0.25 |
| SF36 Role limitations due to physical health | -0.57* | -0.47* | -0.55* | -0.26 | -0.30 | -0.31* | -0.40* | -0.30 |
| SF36 Role limitations due to emotional problems | -0.52* | -0.47* | -0.43* | -0.15 | -0.12 | -0.17 | -0.36* | -0.10 |
| SF36 Energy/Fatigue | -0.79* | -0.67* | -0.58* | -0.19 | -0.35* | -0.41* | -0.49* | -0.28 |
| SF36 Emotional well-being | -0.54* | -0.56* | -0.39* | -0.16 | -0.23 | -0.17 | -0.33* | -0.12 |
| SF36 Social Functioning | -0.70* | -0.63* | -0.57* | -0.26 | -0.35* | -0.38* | -0.53* | -0.22 |
| SF36 Pain | -0.70* | -0.59* | -0.79* | -0.22 | -0.50* | -0.50* | -0.49* | -0.46* |
| General health | -0.70* | -0.55* | -0.67* | -0.30* | -0.45* | -0.49* | -0.49* | -0.30* |
| FACIT-F Fatigue Index (Reversed) | 0.93* | 0.79* | 0.73* | 0.27 | 0.49* | 0.50* | 0.58* | 0.37* |

**Correlations statistically significant at Bonferroni adjusted alpha (m = 72) of 0.00069 (two-tailed)*

^+^ *Data from the SSF-PSS group shows similar results (data not shown).*
